# Data-Analytics Modeling of Electrical Impedance Measurements for Cell Culture Monitoring

**DOI:** 10.3390/s19214639

**Published:** 2019-10-25

**Authors:** Elvira García, Pablo Pérez, Alberto Olmo, Roberto Díaz, Gloria Huertas, Alberto Yúfera

**Affiliations:** 1 Departamento de Tecnología Electrónica, Escuela Técnica Superior de Ingeniería Informática, Universidad de Sevilla, Av. Reina Mercedes, SN, 41012 Sevilla, Spain; elviragruiz@gmail.com (E.G.); pablopg@us.es (P.P.); yufera@us.es (A.Y.); 2 Instituto de Microelectrónica de Sevilla, Universidad de Sevilla (IMSE-CNM-CSIC), Av. Américo Vespucio, 28, 41092 Sevilla, Spain; gloria@imse-cnm.csic.es; 3 R & D Department, Treelogic S.L. 28223 Pozuelo de Alarcón, Spain; rober.diaz@gmail.com; 4 Departamento de Teoría de la Señal y Comunicaciones, Universidad Carlos III de Madrid, Av. De la Universidad 30, 28911 Leganés, Spain; 5 Departamento de Electrónica y Electromagnetismo, Facultad de Física, Universidad de Sevilla, Av. Reina Mercedes, SN, 41012 Sevilla, Spain

**Keywords:** laboratory automation, cell culture monitoring, electrical impedance, data analytics modeling

## Abstract

High-throughput data analysis challenges in laboratory automation and lab-on-a-chip devices’ applications are continuously increasing. In cell culture monitoring, specifically, the electrical cell-substrate impedance sensing technique (ECIS), has been extensively used for a wide variety of applications. One of the main drawbacks of ECIS is the need for implementing complex electrical models to decode the electrical performance of the full system composed by the electrodes, medium, and cells. In this work we present a new approach for the analysis of data and the prediction of a specific biological parameter, the fill-factor of a cell culture, based on a polynomial regression, data-analytic model. The method was successfully applied to a specific ECIS circuit and two different cell cultures, N2A (a mouse neuroblastoma cell line) and myoblasts. The data-analytic modeling approach can be used in the decoding of electrical impedance measurements of different cell lines, provided a representative volume of data from the cell culture growth is available, sorting out the difficulties traditionally found in the implementation of electrical models. This can be of particular importance for the design of control algorithms for cell cultures in tissue engineering protocols, and labs-on-a-chip and wearable devices applications.

## 1. Introduction

The use of artificial intelligence and data analytic methods to model complex biological processes has proved to be a powerful tool that can provide useful solutions in many different application fields [1,2,3]. The electrical cell-substrate impedance sensing technique (ECIS) is a well-established method that allows the real time acquisition of biological parameters (cell growth, motility, activity, or size) of any cell culture and its relationship with the environment through bioimpedance measurements [4]. It can be used in many different studies, such as in toxicity studies, cell growth, cell motility [4,5], cancer progression [6], or stem cell differentiation for regenerative medicine [7].

ECIS is a non-invasive method (it avoids the death of cells along the timeline, overcoming the limitations of end-point protocols). Furthermore, it is a relatively inexpensive technique, as only one sample or petri plate is required for a performance curve. This petri plate includes different electrodes, and the measured voltage between electrodes can be correlated with different biological parameters. It is currently being used in a wide variety of high-throughput laboratory automation data extraction [5,6,7].

The main drawback of the ECIS technique is the need for managing models to decode the electrical performance of the full system, composed of the electrodes, medium, and cells. Several works have been developed in this field. In [4], the impedance was deduced from the electric field equation solution at the cell-electrode interface, giving a three-parameter-based model: the cell-electrode distance, the barrier resistance, and the cell radius. In [8], finite element simulations (FEM) were executed for solving an electrical field considering the whole structure. This method gave one parameter model (Rgap) for describing the gap or cell-electrode interface resistance. In both works, the derived model considered the confluent phase [4] or a fixed area covered by cells [8]. In [9] the model was extended to several cell sizes, allowing one to define the cell-electrode covered area as the main model parameter. A related field to ECIS is electrical impedance tomography (EIT), where the electrical conductivity and permittivity of a part of the body is inferred from surface electrode measurements and used to form a tomographic image of that part. Recent works have shown an interesting improvement on different EIT methods for the decoding of electrical values, with the use of different machine learning methods, such as convolutional neural networks and structure-aware, sparse Bayesian learning [10,11].

In [12], a specific implementation of the ECIS technique was presented based on an oscillation-based-technique (OBT) circuit, wherein oscillation parameters (frequency and amplitude) directly correlate with the cell growth status [13,14]. Specifically, the oscillation parameters were related with the fill-factor parameter of the cell culture, defined as the ratio of the area covered by the cell culture in the electrodes, from 0 (no cells) to 1 (cells fully covering the electrode area), that being an important biological parameter for the characterization of cell growth. In [12], an empirical-mathematical approach was proposed for the calibration and fitting of the cell-electrode electrical models in the OBT circuit. First, a calibration procedure by employing the OBT technique and one specific initial cell density was carried out. Then, results were extended to other assays with the same cell type, but with different cells densities. Using the proposed calibration protocol and the OBT technique, a cell-electrode electrical model for the bioimpedance ECIS technique experiments was obtained for specific cell lines.

In the present work, a new approach was followed, using regression techniques to adjust the electrical measurements to analyze and predict the fill-factor parameter in a cell culture, based on the amplitude and frequency of the signal measured in the same OBT circuit [12]. The proposed approach aims to facilitate the interpretation of impedance spectroscopy data, without the need to establish a complex and often difficult-to-implement electrical model. The final aim was to predict the fill-factor parameter of the cell culture using regression techniques, without implementing the electrical model. The methodology can be extended to any cell type and any cell density, which is what makes it of utmost importance in ECIS experiments and high-throughput laboratory data analysis.

## 2. Materials and Methods

This section describes the technique used for data acquisition (Section 2.1) and the datasets used (Section 2.2) for the implementation of our data analytic model, which is described in Section 2.3. The final Section 2.4, summarizes the experimental procedure followed.

### 2.1. Oscillation-Based Technique

The experiments to be modelled were based on the oscillation-based-technique (OBT) circuit previously presented in [12]. In this circuit, the electrode-cell impedance value (Zcell-electrode) was incorporated as part of a voltage oscillator. The oscillation parameters (frequency of the oscillation or amplitude of the oscillation) were dependent of different biological parameters of the cell culture, such as the fill-factor (percentage of area covered by cells) or the attachment of cells to the electrode surface.

**Figure 1:**
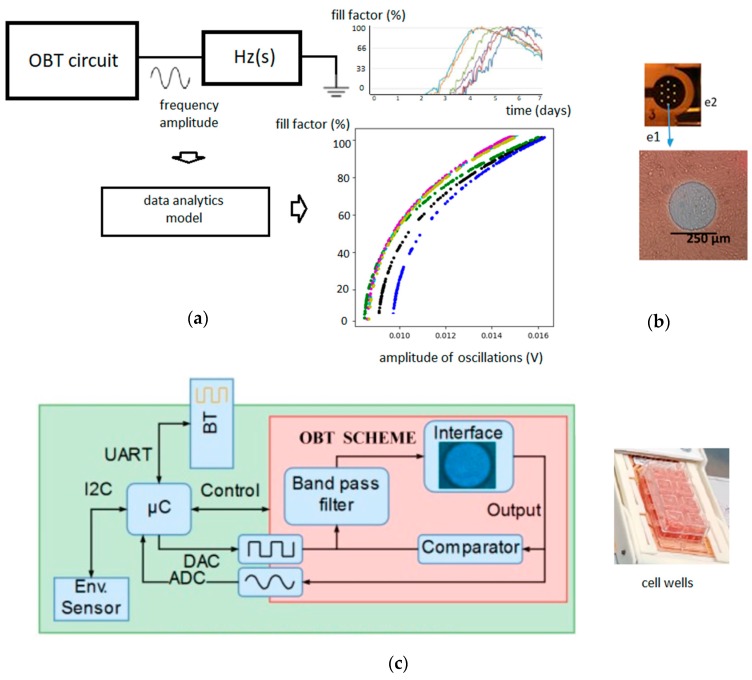
Oscillation-based technique and data-analytics model. (**a**) The oscillation-based technique (OBT) circuit is connected to the Hz(s), the bio-impedance block, which includes the Zcell-electrode. A full description of the OBT circuit is presented in [12]. The frequency and amplitude of each oscillation produced by the OBT circuit are the input parameters for the data-analytics model. The fill-factor of the cell culture and its evolution with the amplitude or frequency of OBT signals is the parameter we want to predict; (**b**) Detail of one of the eight wells of the 8W10E PET cultureware from Applied Biophysics [15] that were used in the experiments, where e1 is one of the 10 circular gold electrodes (the sensing area is the sum of the 10 gold electrodes) and e2 is the reference or ground electrode. Each well has in total an area of 0.8 cm^2^; (**c**) Hardware prototype scheme for the sensor device, used for the wireless monitoring of the cell culture data [14].

With our modeling approach, we tried to estimate and predict the aforementioned fill-factor parameter, based on the measured electrical values of the circuit (amplitudes and frequencies of the oscillations). The use of data-analytic algorithms optimizes the calculation of the evolution of fill-factor with time, based on previous data obtained for the cell line, without the need to resolve complex electromagnetic or electrical models. The Oscillation-Based circuit scheme and data analytics concept used are shown in Figure 1.

### 2.2. Datasets Used

Datasets for two different kind of cell cultures were used in this work. The first one was composed of 40 samples from an N2A cell culture. N2A are a fast-growing mouse neuroblastoma cell line, usually used to study neurite outgrowth, neurotoxicity, or Alzheimer’s disease, among others. This cell line was previously monitored by the OBT and modelled with an empirical-mathematical approach [12]. The same data was used for our new modeling approach.

Secondly, a bigger set of data consisting of six myoblast cell cultures were available to make separate tests of the model designed. Myoblast cell cultures are important for different regenerative medicine applications. To precisely monitor its growth, it is important to control its differentiation process in tissue engineering. This dataset was previously obtained and described in [16].

Every dataset used for this study consisted of three different variables from different cell cultures. Table 1 includes a description of the different variables.

A regression problem was addressed, for which we started from a dataset *D* that contained n training samples in a two-dimensional space of the form:

(1) $$D={\left\{\left(x^{\left(i\right)}|y^{\left(i\right)}\right){|x}^{\left(i\right)}\in {\mathbb{R}}^{2},x^{\left(i\right)}\in \mathbb{R}\right\}}_{i=1}^{n}$$

Our goal was to build a function that can infer the fill-factor as a function of the amplitude and frequency of the measured sine wave.

(2) y=f(x),

### 2.3. Data Processing and Modeling

Different regression models were initially tested. All of these models were implemented and measured using the Python library described in [17]. We got standardized the amplitude and frequency features by removing the means and scaling to unit variance.

#### 2.3.1. Linear Regression Model

This was a linear approach to model the relationship between the fill-factor and the input variables (amplitude and frequency).

(3) $$y=f\left(x_{1},x_{2}\right)=w_{0}+w_{1}x_{1}+w_{2}x_{2},$$

#### 2.3.2. Polynomial Regression Model

A polynomial regression, since we are in a 2-dimensional problem, obtains the following regression function:

(4) $$y=f\left(x_{1},x_{2}\right)=w_{0}+\overset{d}{\underset{i=1}{\sum }}\overset{2}{\underset{j=1}{\sum }}w_{j,i}x_{j\;}^{i}$$

where *d* is the degree of the polynomial.

#### 2.3.3. Regression Trees

Regression Trees (RTs) [18] are a non-parametric, supervised learning method. RTs are built through an iterative process known as binary recursive partitioning that splits the data into partitions or branches, and then continues splitting each partition into smaller groups as the method advances through each branch.

#### 2.3.4. Gaussian Processes

Gaussian Processes (GPs) [19] can learn nonlinear relationships using kernel functions. The prediction is probabilistic (Gaussian) so that one can compute empirical confidence intervals and decide, based on those intervals, whether refitting (online fitting or adaptive fitting) the prediction should be done in some region of interest.

### 2.4. Experimental Procedure

In the N2A cell culture dataset, we randomly selected 90% of data for training and 10% for testing. Since the size of data for testing was small, we repeated the experiments 1000 times and we provide herein, the mean and standard deviation of the mean square error.

In the myoblast cell culture dataset, since we had data from six different cell cultures, we repeated the experiments six times (using five cultures for training and one culture for the test) and we report the mean and the standard deviation of the mean square error.

In every experiment, the hyper-parameters of the algorithm (regularization term in linear and polynomial regression and the depth of the tree in RTs) were selected using a leave-one-out cross validation procedure.

For each algorithm, we considered the prediction of the fill-factor as a function of the amplitude and frequency and the prediction using a single input variable (amplitude or frequency). The mean and standard deviation of the mean square error for each algorithm were calculated and compared.

## 3. Results

### 3.1. N2A Cell Culture

**Figure 2:**
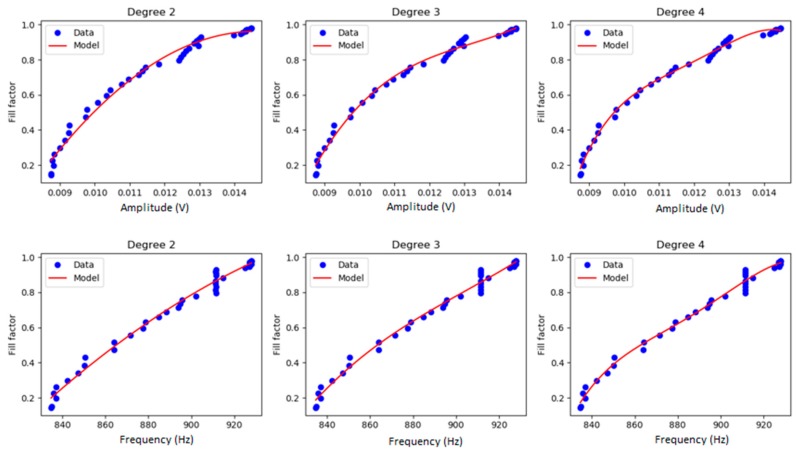
Polynomial regression of the fill-factor against frequency (Hz) and amplitude (volts) for N2A. Different degrees (2, 3, and 4) were used for the polynomial regression, for predicting the fill-factor of the cell culture from the amplitude (volts) and frequency data (Hz).

**Figure 3:**
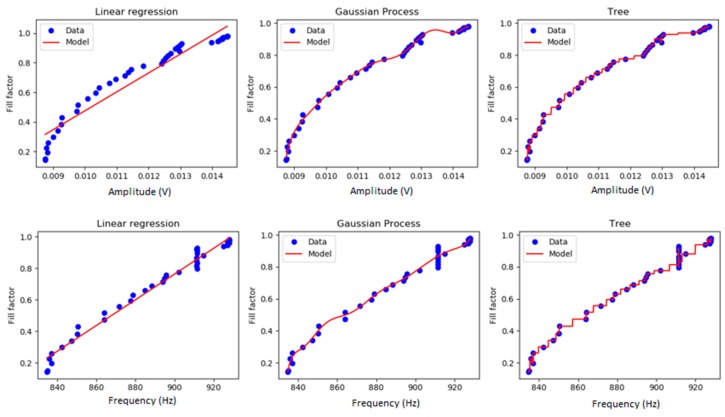
Linear regression, regression trees (RTs), and Gaussian processes (GPs) of the fill-factor against the amplitude (volts) and frequency (Hz) of OBT for N2A. The best results using both variables were obtained using polynomial regression with a degree of four in the polynomial regression. Using a single input variable, the best results were obtained using GPs.

We present here, the results of the N2A cell culture dataset. Figure 2 and Figure 3 show the visual results of the model that predicts the fill-factor against the frequency and amplitude of the circuit signal using the different algorithms and the original samples for N2A. Figure 2 shows the results of polynomial regression using different degrees. Figure 3 shows the results of the fill-factor prediction using linear regression, RTs and GPs.

We observed that a four-degree polynomial regression of fill-factor against amplitude of OBT for N2As and myoblasts succeeded in predicting the fill-factor for this cell culture. On the other hand, GPs obtained the best result for predicting the fill-factor in the case of N2A, compared to linear regression or RTs. Table 2 summarizes, for every algorithm and input variable, the average and the standard deviation of the mean squared error (MSE).

### 3.2. Myoblast Cell Cultures

**Figure 4:**
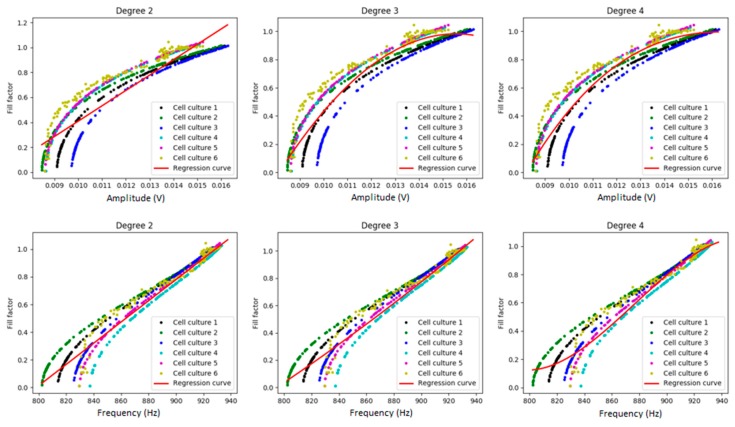
Polynomial regression of fill-factor against frequency (Hz) and amplitude (volts) for myoblasts. We can observe the experimental data obtained for the six different myoblast cell cultures and the regression curve obtained for polynomial regression (red line).

**Figure 5:**
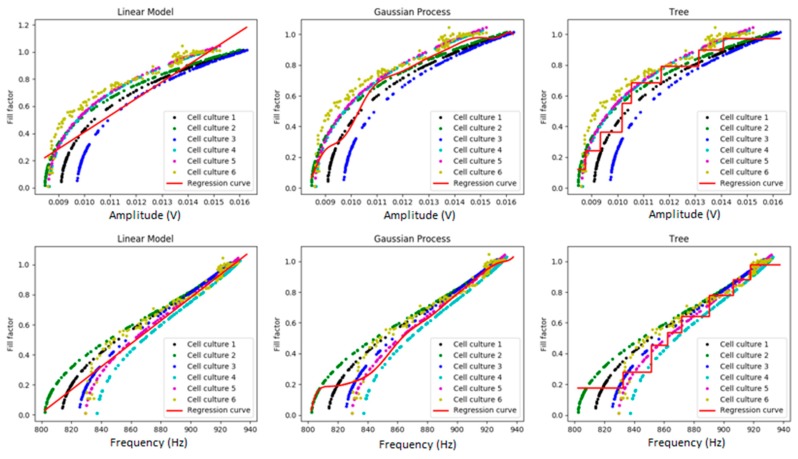
Linear regression, RTs, and GPs of fill-factor against amplitude (volts) and frequency (Hz) for myoblasts. We can observe the model implemented for myoblasts (red line) and the data obtained from the six different myoblast cell cultures.

Figure 4 and Figure 5 show the visual results of the model that predicts the fill-factor against the frequency and amplitude of the circuit signal using the different algorithms and the original samples for myoblast cell cultures. As we can observe, there is a high variance among them for small values of the fill-factor. Figure 4 shows the results of polynomial regression using different degrees, and Figure 5, the results using linear regression, RTs, and GPs.

Table 3 shows the MSEs obtained with the different regression techniques. We observed that in this case, the polynomial regression obtained the best results for the prediction of the fill-factor (with degree four in the bidimensional problem, with degree two for amplitude, and with degree three for frequency).

## 4. Discussion

In this work, a new approach was followed for the interpretation of electrical impedance measurements of cell cultures through ECIS (electrical cell-substrate impedance sensing). ECIS is a well-established method that allows for the real time acquisition of the biological parameters of any cell culture through bioimpedance measurements [4]. It is widely used in laboratories for a wide variety of applications, including tissue engineering, cancer monitoring, and pharmacology [5,6,7]. The main drawback of ECIS technique is the need for managing electrical models to decode the electrical performance of the full system composed of the electrodes, medium, and cells [12,13,14].

A data-analytic technique based on non-linear regression was applied to analyze and predict a specific biological parameter in a cell culture, from specific ECIS measurements. The fill-factor parameter (the ratio of the area covered by the cell culture in the electrodes) was chosen, as it represents an important biological parameter for many different studies, such as cell toxicity or studies of cellular growth [5,6].

A specific ECIS circuit was studied, based on the OBT technique [12]. In this circuit, the electrode-cell impedance value (Zcell-electrode) was incorporated as part of a voltage oscillator. The amplitude and frequency of the OBT measured signal were used as input variables to our algorithms, with the final aim of predicting the fill-factor of the cell cultures. An initial prototype of the circuit is reported, for the wireless and real-time monitoring of cell cultures [14].

Two datasets were used, from N2A and myoblast cell cultures, previously reported in [12,16]. The fill-factor of the cell cultures (the ratio of the area covered by the cell culture in the electrodes) was predicted, based on the electrical values of the circuit measured (amplitude and frequency of the oscillations) and the use of data-analytic algorithms.

Different polynomial regression models were implemented for N2A and myoblast cell cultures: linear and polynomial regression models, regression trees, and Gaussian processes. In the bidimensional problem, a four-degree polynomial regression of fill-factor against the amplitude of OBT for N2A and myoblast succeeded in predicting the fill-factor for this cell culture. When we used a single input variable, GPs obtained the best result for N2A and polynomial regression for myoblast cell cultures. The means and standard deviations of the mean squared errors (MSEs) were studied for all cases.

The results show the appropriateness of the technique in the prediction of the fill-factor in different cell cultures. The data-analytic modeling approach can be used in the decoding of electrical impedance measurements of different cell lines, sorting out the difficulties traditionally found in the implementation of electrical models, provided a representative volume of data from the cell culture growth is available. Unlike in [12], the oscillation frequency and amplitude at the end of the experiment is not needed in advance to decode the electrical values, enabling this method to perform actual, real-time measurements of the fill-factor parameter. The technique can be of use for the prediction of the fill-factor in the real-time monitoring of cellular processes [4,5,6,7,12,13,14] and can be of particular interest in the design of control algorithms.

In a practical sensing problem, the specific setups would need to be off-line calibrated, with the performance of different experiments, to characterize the electrical measurements for a specific cell-line, the specific substrate, and the monitoring circuit. However, once characterized, the proposed method provides an important added-value for the real-time decoding of electrical impedance measurements and in the automatization and processing of biological data.

The proposed algorithms could be extended for the prediction of new biological parameters, and in general, for the improvement of high-throughput data analysis in laboratory automation. Similarly to [10,11], where convolutional neural networks and structure-aware, sparse Bayesian learning were used to improve EIT measurements, our results show the potential of machine learning methods to interpret electrical impedance measurements in cell culture monitoring. In our case, with the available data (frequency and amplitude), the direct and straight-forward statistical models used were considered optimal for the prediction of the fill-factor, although other machine learning methods like the ones proposed in [10,11], could be used if we have other interesting input data, such as cell culture images.

Future, specific applications of the technique could be found in the control of biological processes in tissue engineering, in order to provide the needed control signal in cellular differentiation processes [16]. Additionally, it could be used in the process of control of different lab-on-a-chip or wearable systems, where it would be necessary as a real-time signal to control the actuation system [20]. The method can be used, in general, for the improvement of the utilization of ECIS in smart sensor systems in the field of healthcare, where the real-time and rapid analysis of high-throughput data is essential.

## Figures and Tables

**Table 1:** Description of electrical and biological parameters.

Variable	Description
x_1_	Amplitude of the sine wave generated by the OBT circuit and Hz(s) ^1^
x_2_	Frequency of the sine wave generated by the OBT circuit and Hz(s) ^1^
y	Fill-factor corresponding to the amplitude and frequency values. It’s the parameter to predict, which can be compared with experimental data in [12,16].

^1^ Data was obtained from the experiments reported in [12,16].

**Table 2:** Mean and standard deviation of the mean squared error (MSE) using the different techniques, for N2A cells.

Regression Model	MSE and Standard Deviation		
	Bidimensional	Amplitude	Frequency
Linear Regression	1.41 × 10^−3^ (1 × 10^−3^)	6.55 × 10^−3^ (3.45 × 10^−3^)	1.76 × 10^−3^ (1.1 × 10^−3^)
Polynomial Regression (*d* = 2)	3.3 × 10^−4^ (3.4 × 10^−4^)	1.2 × 10^−3^ (1 × 10^−3^)	1 × 10^−3^ (7.9 × 10^−4^)
Polynomial Regression (*d* = 3)	1.6 × 10^−4^ (1.6 × 10^−4^)	7.9 × 10^−4^ (6 × 10^−4^)	1.1 × 10^−3^ (7.4 × 10^−4^)
Polynomial Regression (*d* = 4)	8.44 × 10^−5^ (1.1 × 10^−4^)	5.7 × 10^−4^ (3.3 × 10^−4^)	9.45 × 10^−4^ (6.9 × 10^−4^)
Regression Trees	9.78 × 10^−4^ (6.9 × 10^−4^)	1 × 10^−3^ (5.7 × 10^−4^)	2.2 × 10^−3^ (1.39 × 10^−3^)
Gaussian Processes	1.6 × 10^−4^ (3.4 × 10^−4^)	4.34 × 10^−4^ (3.4 × 10^−4^)	1 × 10^−3^ (7.2 × 10^−4^)

**Table 3:** Means and standard deviations of the MSEs using the different techniques, for myoblasts.

Regression Model	MSE and Standard Deviation		
	Bidimensional	Amplitude	Frequency
Linear Regression	4.7 × 10^−3^ (2.5 × 10^−3^)	1.3 × 10^−2^ (4.8 × 10^−3^)	5.9 × 10^−3^ (3.8 × 10^−3^)
Polynomial Regression (*d* = 2)	5.1 × 10^−3^ (3.7 × 10^−3^)	8.4 × 10^−3^ (6.8 × 10^−3^)	6 × 10^−3^ (3.7 × 10^−3^)
Polynomial Regression (*d* = 3)	2.4 × 10^−3^ (1.5 × 10^−3^)	9 × 10^−3^ (8.3 × 10^−3^)	4.9 × 10^−3^ (3 × 10^−3^)
Polynomial Regression (*d* = 4)	1.2 × 10^−3^ (8 × 10^−4^)	9.5 × 10^−3^ (8.8 × 10^−3^)	5.1 × 10^−3^ (3.2 × 10^−3^)
Regression Trees	7 × 10^−3^ (3.8 × 10^−3^)	1.3 × 10^−2^ (1 × 10^−2^)	6.6 × 10^−3^ (3.3 × 10^−3^)
Gaussian Processes	4.1 × 10^−3^ (5.2 × 10^−3^)	1.4 × 10^−2^ (1 × 10^−2^)	6.1 × 10^−3^ (3.8 × 10^−3^)
